# A review of organizational arrangements in microfinance and health programs

**DOI:** 10.29392/joghr.2.e2018024

**Published:** 2018-10-01

**Authors:** Jenny Ruducha, Meena Jadhav

**Affiliations:** 1 Braintree Global Health, Cambridge, Massachusetts, USA; 2 The India Nutrition Initiative, Lucknow, Uttar Pradesh, India

## Abstract

**Background:**

Combining health programs with microfinance is gaining more recognition as a pathway for improving health and increasing access to health services among the poor, especially women living in low-income countries. Recently published reviews have summarized the changes in health behaviors and health outcomes due to the effective layering of health interventions with microfinance initiatives. However, a large gap remains in defining and understanding the organizational strategies for implementing effective health programs and services that improve the health and social well-being of women and their families.

**Method:**

As microfinance organizations and the global health community recognize the largely untapped potential of developing effective multidimensional channels of providing access to a variety of health interventions through a microfinance platform, there is a need for more evidence to guide organizational strategies that are feasible, sustainable and produce results. We developed a framework and classification scheme for identifying organizational arrangements between microfinance and health, outlined the criteria for article identification and selection, and reviewed original articles that included a discussion on organizational strategies published in peer-reviewed journals to better inform future research and effective program development.

**Results:**

Our review found that most MFIs operate in cooperative and collaborative partnerships for expanding health and social services with health education as the leading intervention. The extreme ends of the integration-partnership continuum, ie, no partnership on one end and complete merger on the other, are rare if they exist.

**Conclusions:**

The drivers of organizational strategy are related to the context, health needs of the clients, and individual capacities of MFIs to develop effective services. However, approaches to establishing these processes and decision-making for effectively structuring and delivering health and microfinance services is an inadequately explored area. Future progress depends on bridging public health, microfinance, and organizational research silos to study how different organizational arrangements affect implementation and outcomes.

The expansive reach of microfinance to more than 200 million households globally through more than 3,000 MFIs (*1*) makes it a large-scale platform to reach the poorest of households across the globe with basic health and social services. Implemented in its full form, microfinance institutions typically include financial and credit services but have increasingly expanded their services to include health education, health care, health insurance, education and linkages to other services (*2*).

To carry out their expanded scope, MFIs are part of a growing trend of multi-organizational and cross-sectoral partnerships to address complex social and health problems that exceed the management and implementation ability of any one organization (*3*). The purpose of our review is to apply a new organizational arrangements framework to analyze the methods that have been utilized for implementing specific types of health services and products. While recent reviews have summarized the relationship of health programs linked to income generation in poor communities, on a number of diverse health behaviors and outcomes, the organizational arrangements have not received much attention.

Recent studies and reviews citing positive effects on health indicators include: HIV-related outcomes (*4*); behavior change for HIV prevention (*5*); women’s health (*6*); and health knowledge, health behaviors related to fertility, morbidity, gender-based violence and utilization of health services (*2*). However, these results are limited and depend on the type of program, sustainability of MFIs and contextual conditions (*5*). Additionally, the mechanisms linking microfinance to improved health remain largely unknown due to lack of specific descriptions and analysis (*6*) and the processes underlying organizational arrangements that contribute to these outcomes have not been examined.

As more MFI’s recognize the necessity of offering multidimensional services as a pathway for poor families to come out of poverty and improve health (*2*) there is a strong need to study and develop evidence-based, sustainable and feasible implementation approaches (*7*, *8*). Public health studies are typically focused on outcomes with few details of the organizational structures and processes while organizational studies provide theoretical frameworks and strategy formulation but do not correlate these findings with outcomes (*9*). Effective implementation will require a thorough understanding of the organizational strategies and arrangements and the relationship to outcomes. Our review begins to fill this gap by applying an organizational arrangement framework to existing studies to identify the range of integrated and partnership approaches to implementing multi-sectoral services. Our aim is to contribute to a better understanding of the types of organizational strategies that may guide the future designs for scaling-up microfinance and health-related services. Future progress can be made by bridging public health, microfinance, and organizational research silos to develop standard terminology, frameworks, and methods for studying how different organizational arrangements affect implementation and outcomes to inform program development.

## REVIEW METHODS

**Figure 1:**
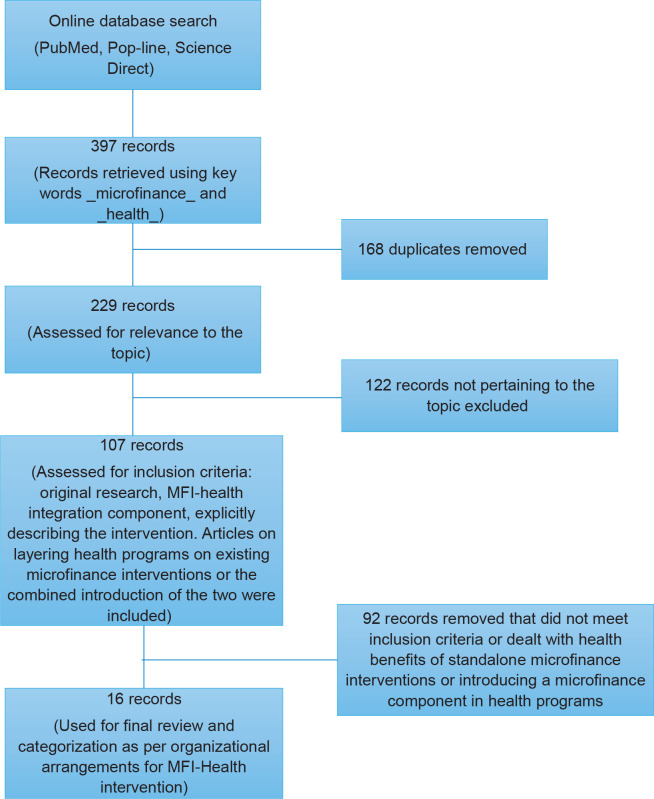
Literature search process

We reviewed the published literature in English using online PubMed, Science Direct, and Popline databases from the year 2000 to 2016. The key words “microfinance” (and) “health” that appeared anywhere in the title or abstract were used in the advanced search features to identify articles. We limited our key search words to “microfinance” and “health” attempting to include programs or interventions that were comparable. Our focus was to review the published research containing strategies that have been rigorously evaluated and therefore providing evidence-based recommendations for organizing health programs in microfinance institutions. We included studies that focused on access and delivery of different health programs with microfinance and explicitly described the intervention, were full-text articles based on original research and published in peer-reviewed journals. Studies that focused on health outcomes of microfinance as a stand-alone intervention or dealt with microfinance as an intervention tool in existing health programs were excluded. Articles that explicitly described the institutional arrangements and processes of providing direct services or forms of linked health services and products through microfinance organizations and partnerships were used in the final analysis (**Figure 1**).

We categorized the articles by modifying three approaches used in earlier reviews – based on institutional arrangement, health theme, and health service type (*2*, *7*, *10*, *11*). We maintained the health theme and health services components but reformulated the institutional arrangements to further clarify the organizational mechanisms for enabling a multidimensional approach to poverty alleviation through the provision of health and financial services. As the source for the primary synthesis of the articles, we teased out the institutional arrangements to classify the articles into the following approaches: integration and partnerships. In contrast to earlier formulations, that use “integration” more generally to include both unified and parallel as well as linked integration (*7*), we make a distinction between integration and classify linked approaches as partnerships. In streamlining the terminology used by multiple disciplines, we define integration as when an MFI delivers both financial and health services or benefits directly through its own organization. This may exist when the MFI develops more internal skills and resources that may include health education and other products and services related to improving health or other related outcomes. A partnership exists when an MFI agrees to work with one or more organizations, such as government, NGO, or private providers for a specific purpose such as to enable access to health programs or health-related products or services.

Previous literature has identified three types of partnership models − cooperative, collaborative, and integrated that run along a continuum of coordination arrangements. The basic cooperative model applies when each partner remains autonomous in budgeting, staffing, and decision-making. In a collaborative model, there is more sharing of resources, decision-making, and accountability. An integrated model of partnership is when there is mixing of resources and a surrender of individual autonomy to a new entity for decision-making (*12*). Due to the varying use of the terms integration and partnerships in the literature describing institutional arrangements between MFI’s and health program delivery vehicles, we chose to classify the articles according to our definition of integration and partnership. We adapt the types of partnership models identified by our literature review, to create a unidirectional continuum of organizational arrangements that range from no partnership to complete merger or unification of the MFI and health-related organization. From the perspective of the MFI and for the purpose of our paper we term the ‘no partnership’ end of the continuum as integration under which the MFI incorporates health functions into its institutional portfolio with no involvement of an external health organization. To help distinguish this internal MFI integration from the third partnership type, which is also named integration in the original source, we rename the other end of the continuum as ‘unification’ but retain its original definition as a merger between two organizations.

**Figure 2:**
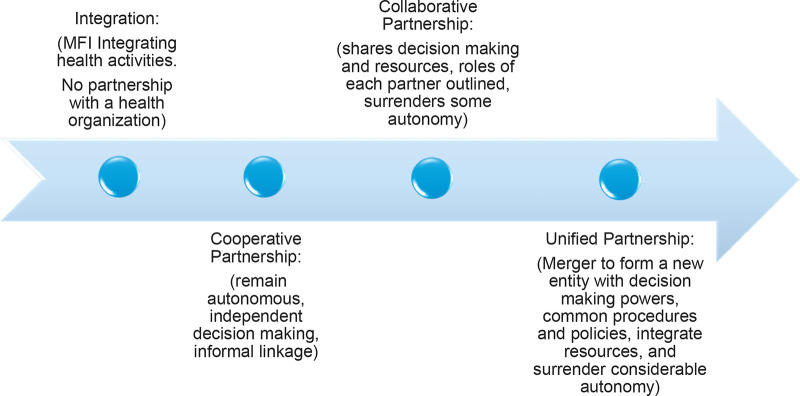
The MFI-Health integration-partnership continuum. Partnership types adapted from The Partnership Tool Kit, Collaboration Roundtable, 2001 (*13*).

The partnership continuum is adapted from the partnership toolkit developed by the Collaboration Roundtable (*12*) and modified to accommodate the categories of organizational arrangements for MFI-Health programs, used in this review (**Figure 2**). The MFI may decide to diversify its portfolio and take on the role of a health organization or partner with an external health organization to support its health initiatives. Depending on the approach, the organizational arrangements for delivery of microfinance-health programs may fall anywhere on a continuum from no partnership to complete merger or unification of MFI and health organizations. Theoretically, or in the long run, the integration-partnership continuum of organizational arrangements between an MFI and health organization can be bi-directional. An MFI may initially enter a cooperative or collaborative partnership with a health organization and eventually integrate the health program delivery into its institutional profile and function autonomously. For the sake of clarity and alignment with the main purpose of the paper to review and classify the organizational arrangement employed by MFI-health interventions, we keep this integration-partnership continuum as unidirectional. It is based on degrees of partnership ranging from no partnership to complete merger. To provide further clarity for our review, the different categories of organizational arrangements have been defined in **Table 1**.

**Table 1:** Definition of categories of organizational arrangements for MFI-health programs

TYPE OF ORGANIZATIONAL ARRANGEMENT	DEFINITION FOR THE PURPOSE OF THIS PAPER
**1. Integrated**	Under an integrated approach, a microfinance organization diversifies its portfolio and initiates a health program for its clients, completely on its own, without the support or involvement of any external health organization.
2. Partnership*	Partnerships refer to organizational arrangements under which a microfinance organization formally or informally ties up with a health organization to deliver health services to its clients. Depending on the extent of partnership it can fall anywhere between informal cooperation, significant collaboration, to a complete merger or unification with the health organization.
**a) Cooperative**	Cooperative partnerships are under which the MFI takes the support of a health organization under an informal contract for delivering the health service. The two organizations remain autonomous with each retaining its decision-making responsibility, staff and budget, and separate identity.
**b) Collaborative**	Under collaborative partnerships, the MFI and Health organization shares decision making responsibility and authority, has particular roles and responsibilities, is accountable to the other, contributes resources, and surrenders some measure of its autonomy.
**c) Unified**	Unified partnerships are when the two organizations become an integral part of each other or create a separate unified entity with decision-making powers, integrate resources, have common policies and procedures, and surrenders a considerable amount of autonomy.

* Partnership definitions adapted from - Collaboration Roundtable. The Partnership Toolkit : Tools for Building and Sustaining Partnerships Collaboration (13).

### Conceptual framework

**Figure 3:**
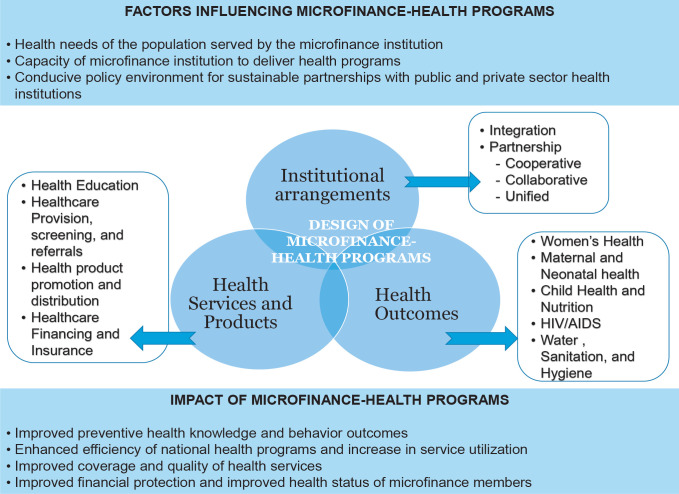
The conceptual framework for the review.

The conceptual framework for the review captures the structural and functional aspects of designing MFI-health combined programs and show how they are driven by contextual factors affecting the institutional arrangements, health services and products, and outcomes (Figure 3). Health needs of the population served by the MFI, capacity of the microfinance institution to deliver a health program and an enabling environment for the MFI to form and sustain partnerships with public and private sector health institutions are the key contextual factors influencing the design of MFI-health programs. These factors are influential in deciding the type, scalability, and replicability of MFI-health interventions. Depending on the health needs of the clients, the health theme addressed by the MFI could span a diverse set of interventions such as women’s health, maternal and neonatal health, child health and nutrition, HIV/AIDS, water, sanitation and hygiene. At the same time, individual capacities of the MFIs influence the complexity and scale of the interventions. MFIs with poor capacities may limit their interventions to health education while strong MFIs expand their health services to include higher functions like providing health care, promoting health products, and financing health care. The capacity of the MFI along with the opportunity and availability of health organizations to join in partnership, and the scale and complexity of the health services or products, plays a role in deciding the type of institutional strategies. They can range from integration (MFI integrates health function in its services with no partnership with a health organization) to cooperative, collaborative or unified partnerships. Cross-sectoral interventions involving MFIs and public and private sector health institutions have the potential to impact health knowledge and behavioral outcomes, increase coverage and quality of health services, complement financing of healthcare among the poor and vulnerable population with a high burden of ill-health and thus improve the efficiency of the health sector.

## RESULTS

The individual articles included in our review are synthesized using a framework that teases out organizational arrangements for delivery of the interventions to classify them as – Integrated, or Partnerships. We present the articles along with their health themes and services, but the focus is on understanding the organizational arrangement used for microfinance-health combined services. An analytical look at these organizational arrangements provides insights on the choice of organization strategy used by the MFI based on their context – setting, client health needs, the scale of operations and financial sustainability. The challenges and enabling factors for the MFI-Health organization briefly mentioned in the discussion section of some articles, provide valuable insights for deciding on the appropriateness, feasibility, scalability, and replicability of the MFI-Health interventions in other settings. These challenges, as well as enabling factors, are discussed separately after the findings section. We summarize the articles (**Table 2**) and further present individual studies, classified along the integration-partnership continuum of MFI-health combined interventions. Please refer to the online supplementary (Table S1 in **Online Supplementary Document**) for complete details of these articles.

**Table 2:** Organizational arrangements based on the health service type

STUDIES WITH ORGANIZATIONAL ARRANGEMENTS ON A PARTNERSHIP CONTINUUM	HEALTH EDUCATION	HEALTH CARE PROVISION	PRODUCT PROMOTION	HEALTH SCREENING	HEALTH FINANCING	REFERRAL AND LINKAGES
***Integrated Organization arrangements:***						
Integrated						
No example						
*Integrated with a cooperative component*						
Hamid et al, 2011 (*13*)		Yes		Yes	Yes	Yes
Geissler and Leatherman, 2015 (*14*)	Yes	Yes		Yes		Yes
***Partnership Organization arrangements:*** **Cooperative partnerships:**						
Carothers et al, 2009 (*16*)	Yes				Yes	
Leatherman et al, 2013 (*17*)	Yes	Yes			Yes	Yes
Witte et al, 2015 (*15*)	Yes					
Saha et al, 2015 (*18*)	Yes	Yes			Yes	Yes
***Collaborative partnerships:***						
Pronyk et al, 2006 (*19*)	Yes					
Seiber and Robinson, 2007 (*28*)					Yes	
Pronyk et al, 2008 (*21*)	Yes					
Davis et al, 2008 (*26*)			Yes			
De La Cruz et al, 2009 (*23*)	Yes		Yes			Yes
Freeman et al, 2012 (*26*)	Yes		Yes			
Flax et al, 2014 (*22*)	Yes					
Spielberg et al, 2013) (*21*)	Yes					
Christoffersen-Deb et al, 2015 (*24*)	Yes	Yes				Yes
**Collaborative/Unified partnership:**						
Bannerjee et al, 2014 (*28*)					Yes	
*Unified partnership*						
No example						

### Integrated organizational arrangements

As detailed in the methods section we define integrated institutional arrangements when an MFI delivers the health service or benefit directly through its own organization. We could not find any study with a pure integrated approach where the MFI did not partner or link with an external organization. Though in practice, MFIs that deliver informal health education through group meetings may do so with an integrated approach without any external support.

Some large microfinance institutions such as Grameen Bank in Bangladesh and Pro Mujer in Latin America have used a hybrid approach of integration with a cooperative component to successfully deliver clinical services alongside microfinance activities, though the services were restricted to health screenings, and basic health services. They incorporated a cooperative partnership arrangement with external health providers for referrals to higher levels of care. The Grameen Bank implemented a Micro Health Insurance (MHI) scheme to provide healthcare directly to their clients by establishing health centers along with paying for their coverage. The bank sold an annually renewable prepaid insurance card to the poor, both members and non-member, of the MFI with the delivery of curative services at reduced medical consultation fees, discounts on drugs and test, hospitalization benefits, and free annual health checkup and immunization (*13*). Another MFI in Latin America, Pro Mujer, fully integrated clinical service delivery alongside microfinance services through their universal screening program for Non-Communicable Diseases (NCDs) and provision of primary care services. The universal screening program included free health screenings (body mass index, blood pressure, clinical breast examination and blood sugar level) but Pap smears were provided at nominal cost. A unique feature of this intervention was co-location of health education and clinical services along with mobile clinics for remote areas. Health education was provided by trained credit officers. The cost of these services was covered by interest charged on microfinance loans (*14*).

### Cooperative partnerships

MFIs often engaged in cooperative partnerships with health-specific organizations for the development of training curriculums for health education, training of trainers, linking with national health programs, referrals to external healthcare providers and organizing health camps in MFI areas. Under a cooperative partnership, MFIs utilize the expertise of health organizations for a one-time activity such as training of credit officers or for a specific component of the MFI-Health intervention such as referral for higher levels of care.

In Mongolia, an HIV and sexual risk reduction curriculum was successfully delivered alongside a savings-led microfinance program among sex workers leading to a reduction in unprotected vaginal sex and a reduction in the number of paying partners. The Gender and Entrepreneurship Together curriculum designed by the International Labour Organization and the Global Financial Education program by Microfinance Opportunities (Washington, DC), was adapted for preparing the training curriculum (*15*).

A unique intervention in Egypt enhanced safety for children working in small businesses funded by microfinance loans by providing training on workplace safety for children, hazard assessment and mitigation training to loan officers. Business owners committed investments in child occupational health and safety through an inbuilt loan disbursement mechanism by increasing the loan amount. The intervention was originally developed as a cooperation between loan officers, microenterprise owners, and working children, but allows loan officers to withhold future loans if business owners fail to deliver on agreed improvements for working children (*16*).

MFIs in Bolivia, Burkina Faso, and Benin offered health loans, health savings account, and health loans linked to savings accounts to their clients providing protection from financial risk for health care costs. These loans were charged at the lower interest rate, had flexible and longer repayment periods, and in some cases were paid directly to health providers to ensure their use for a health purpose. Within two years of initiation, 1% of the MFI clients (6% as per authors’ calculations) had received health loans (*17*).

In India, voluntary health workers were nominated by two SHGs to raise awareness on maternal and child health issues, hygiene and sanitation. Microloans were provided for constructing toilets, and health insurance was provided by the MFI. The health services were delivered through mobile and stationary health camps, organized by MFI for promoting these services along with referrals to external health providers in case of danger signs of pregnancy or child health complications. SHG women receiving the health program had higher odds of delivering their babies in an institution, feeding colostrum to their newborn, and having a toilet at home. No statistically significant reduction in diarrhea among children was found. There was also no decrease in out of pocket health expenditure even with health insurance provided (*18*).

### Collaborative partnerships

Most studies fall into this category, though the level of collaboration may vary. In South Africa, a participatory learning and action curriculum called Sisters-For-Life (SFL) was integrated into loan meetings and delivered through a separate training team. Microfinance services were implemented by the Small Enterprise Foundation (SEF), Tzaneen, South Africa. The intervention also involved a phase of wider community mobilization through partnerships with local institutions along with the establishment of committees targeted at intimate partner violence such as crime and rape. The intervention led to reductions in the level of intimate partner violence and thereby the risk of HIV (*19*). A further evaluation of the intervention found reductions in HIV risk behavior among the participants (*20*). In West Bengal, India, adolescent girls, and their mothers were enrolled in a non-formal education program called Learning Games for Girls (LGG) through the MFI platform. MFIs were trained in non-formal health education methods through Reach India, a private sector franchise involving a network of two-person teams that train self-help promoting institutions. The training included savings, hand-washing, diarrhea prevention, nutrition, sexual and reproductive health, and HIV/AIDS. The training led to significant gains in HIV knowledge, awareness that condoms can prevent HIV, self- efficacy for HIV prevention and confirmed use of clean needles (*21*). In Nigeria, credit officers integrated learning sessions for promoting national breastfeeding recommendations into loan meetings along with participant generated songs and drama at these meetings, followed by weekly text and voice messages to a cell phone provided to each group. The intervention was originally developed by self-help groups worldwide and implemented by Partners for Development, a USNGO in collaboration with four local community-based organizations. IEC material from the ministry of health in the form of posters and leaflets were also distributed in loan meetings. The study found increased adherence to breastfeeding recommendations — exclusive breastfeeding, timely initiation of breastfeeding, and feeding of colostrum (*22*).

Some integrated interventions also included access to health products along with health education as part of their design. In Ghana malaria education modules developed by Freedom from Hunger (FFH) were integrated into MFI loan meetings delivered by field agents. The field staff was trained in collaboration with national malaria control programs and health professionals. The MFI was linked to distribution networks of ITNs and antimalarial providers to ensure access to these products. The intervention led to the enhanced knowledge of malaria prevention and use of ITNs among pregnant women (*23*). In Kenya, the Academic Model Providing Access to Healthcare (AMPATH), in partnership with the Government of Kenya, launched a peer support model, grouping women at the start of their pregnancies. The platform of social fundraising well known to the women was used to form mother-child investment clubs. The women’s group meetings were utilized by community health workers to disseminate health information, organize referrals, and build relationships with women. The intervention led to improved health behaviors and care-seeking during pregnancy and infancy with an increase in prenatal visits, exclusive breastfeeding, and home visits by community health workers with reduced instances of stillbirths and newborn deaths (*24*). A study in Hyderabad city of India, using a hypothetical readiness approach to microloan programs based on actual WaterCredit program by WaterPartners International found that a substantial proportion of poor households were willing to invest in a water and sewer network connection if provided with micro-loans, even at a commercial rate of interest. The actual intervention would have further required substantial collaboration with the government water and sewer connections department to execute (*25*). In yet another instance of collaborative partnership, ACCESS development services, a support organization for an alliance of MFIs partnered with Hindustan Lever Limited (HUL), a water filter manufacturer to promote drinking water safety by providing micro-loans to purchase the water filters. The intervention found an increase in water quality among the adopters but low uptake among the poorest who needed it the most. Also, among the adopters correct and consistent use was a challenge due to low awareness of need, access and affordability of the replaceable battery (*26*).

In a unique intervention of collaboration with private providers, micro-loans were provided to small private sector healthcare providers in Kampala, Uganda to use as working capital to purchase drugs or equipment, or to renovate or upgrade their clinic. The study found improvement in the perceived quality of care among clients especially due to increased drug availability (*27*).

### Unified partnerships

There were no examples in our review of a complete unified type of partnership between any MFI and health organization. However, we found one example of a hybrid (unified and collaborative) partnership model. SKS, an MFI in India, partnered with ICIC-Lombard, a private insurance company, to launch a bundled mandatory health insurance product along with microfinance loans. Clients had the option to seek care from various approved health facilities for cashless treatment or pay out-of-pocket at other facilities to be reimbursed later. The policy only covered hospitalization and maternity expenses. The MFI was also involved in administering enrollment and initial processing of claims while the private insurance company provided back-end insurance. The uptake of the insurance product was low due to low insurance demand in the community. Microfinance clients were even found to give up microfinance to avoid purchasing health insurance. Later the product was made voluntary but led to a breakdown of the partnership due to this unilateral decision by the MFI (*28*).

## DISCUSSION

The MFI global platform has the potential to improve livelihoods of the poor and reach households with health messages, referrals, and other health-related services. In our review, most MFIs engage in cooperative and collaborative partnerships with health organizations for expanding social, health, and capital resources. The extreme ends of the integration-partnership continuum, ie,, no partnership on one end and complete merger on the other, are rare if they exist. A primary driver for partnership may be the need to access key resources that are lacking or insufficient at the individual organization level. Such assets require the hard resources of money and materials, as well as important soft resources, such as managerial and technical skills, information, contacts, and credibility/legitimacy (*29*).

Almost all organizational approaches that link health and microfinance in the review have shown to be successful in at least some aspects of improving health behaviors and related outcomes. Health education carried out during loan meetings is the most common low resource strategy adopted by most MFIs. This is supported by earlier research that has shown that incorporating health education into microfinance activities to be a cost-effective and sustainable organizational arrangement (*30*). National health education programs that depend on community-based action for their success could bank on organized community groups including the SHG groups to expand their reach and effectiveness. The platform of group meetings can serve as a sustainable communication channel between local government health officials and the community. Such a mechanism can be particularly useful in countries where government health promotion programs are generally delivered in campaign modes and require wider community mobilization and participation. Women are the primary caregivers of the family and therefore the platform of women SHGs could serve as a channel to reach adolescent girls, youths and men for health programs specifically targeting such subpopulations. The frequent interface of the same women of a microfinance group provides the unique opportunity to leverage women groups for reinforcing health messages for behavior change. This opportunity is often missed in health education approaches using mass-media strategies where the message recipients are generally not available for follow-up.

MFIs providing direct health care services, health screenings, and referrals, though few show the potential contribution of MFIs in increasing health access for the clients they serve. Depending on the context, alternative approaches have been used to provide healthcare through MFI owned health facilities, outreach clinics at loan meetings, and mobile and stationary health camps in the community. MFIs, in many instances, are in a privileged position to maintain a permanent relationship with communities based on trusting relationships that have been identified in the international literature as a key component of effective partnerships (*31*). This position can be used to attract partners for expanding the healthcare service coverage. However, the shortage of locally available health providers can be a difficult barrier to overcome.

Few MFIs are involved in population-based screening programs especially targeting women of reproductive age such as for breast and cervical cancer screening and other NCDs. Provision of such specialized health services would require additional resources. Not all MFIs have the capacity (either operational or financial) and commitment to launch health interventions that impact outcomes (*21*). Many MFIs are highly leveraged as the loan-toasset ratio hints that MFIs have their assets tied mainly to the lending business (*32*). A rise in the financial expense ratio may induce MFIs to broaden their service scope (*32*). Though large MFIs were found to successfully add health care delivery to their interventions, such examples are few. Studies have shown that financial productivity can ensure better social outreach productivity if it is effectively channeled as they are complementary to each other (*33*, *34*). Moreover, understanding the need for productivity will allow MFIs to self-improve and help merge any possible gaps between financial sustainability and social outreach (*35*).

Most MFIs in our review did not provide healthcare services themselves but were instrumental in building partnerships for providing health education, health screening, insurance, referrals and access to health products and health services. Partnership is a dynamic relationship among diverse actors and organizations based on mutually agreed objectives through a shared understanding of the most rational division of labor based on the comparative advantage of each partner (*29*). It encompasses mutual influence, with a careful balance between synergy and respective autonomy, which incorporates mutual respect, equal participation in decision-making, mutual accountability and transparency (*29*). The presence of an “enabling structure” such as brokering or mediating organization is seen as a key factor in facilitating action (*36*). On the partnership continuum, cooperative or collaborative or a hybrid may depend on the scale of the program, type of program, and need for ongoing support to the MFI. In complex social interventions with uncertainty about how to achieve certain outcomes, some assert that more formal standards and pre-existing procedures are necessary whereas others argue that nonprofits are more likely to use informal coordination mechanisms and fewer formal controls than businesses or governmental entities (*37*).

It is also important to identify contexts and areas where microfinance groups may be the best available platform or have significant potential to contribute to parallel local and national efforts. While making informed choices based on existing evidence, an understanding of contextual factors including the settings, characteristics of client population, operational and financial capacity of the MFI, availability and type of partnership opportunities, may help the MFI to choose the most appropriate organizational arrangement for combining MFI-health services. In one study, health provisioning by an MFI was found to be effective in places where government health facilities were not functioning well. The author goes one step further and proposes that government should contract out poor functioning health centers to be run by MFIs, optimizing resources and avoiding duplication (*13*). In places, with well-functioning public health systems, optimal use of available resources by partnering with health or government organizations could help microfinance institutions to expand their basket of health services and achieve sustainability.

Challenges to the implementation of microfinance-health programs exist across different organizational arrangements. In cooperative partnerships involving referrals to external health providers, distance to the MFI health center could also be a barrier (*13*) or tracking referrals made to higher facilities due to weak linkages with external providers (*14*). Financial constraints can result in problems delivering interventions such as suspending learning sessions due to repayment problems among the microfinance groups (*23*). Most MFIs are also limited in record keeping capacity to monitor the health interventions such as maintaining records of drug stocks or products distributed (*26*, *27*), as collaborative partnerships require even more investment of time and other resources. Lack of clarity in defining professional boundaries, reconciling different accountability structures and diffusely articulated goals can further undermine collaborative partnerships (*38*–*41*). Further challenges on the collaborative-unified partnership continuum include lack of transparency, procedural delays such as in reimbursement of claims, and trust issues (*28*).

The lack of any examples in our review of standalone MFI organizational integration of services points to the major challenge of initiating and sustaining MFIs that provide multi-dimensional services. In MFI-Health integrated interventions, the continuity of the health activities depends on the solidarity and continuity of microfinance groups. To address this issue, some health interventions are designed around savings-led microfinance, ie, the health services and benefits are contingent upon the beneficiary accumulating certain minimum individual savings (*17*, *25*). While this helps sustainability by financing the health intervention, it is a major deterrent for the poorest population with no capacity of savings (*25*). The challenges due to the arrangement or type of microfinance services affecting stability or community acceptance of MFI services may indirectly affect the continuity of social and health services layered on such microfinance services. Micro-loans that involve monthly payments (besides interest) are found to have less acceptability. Opening a savings account requires a personal identification document not available in some rural communities. Also, most rural banks have group savings and not individual savings (*17*). Formation of joint liability groups was found to be a requirement for availing individual loans in some cases and was cited as one of the reasons for non-participation by clients (*25*). Similarly, health services provided by MFI that charge consultation fees (copayments) could deter some to avail care, especially in communities where the use of informal health care is high. Interventions that depend on voluntary work by SHG members may suffer from a lack of motivation where the volunteers are paid no honorarium or where the payments were not to their satisfaction (*18*).

Attention needs to be paid to the distinctive challenges of establishing and sustaining partnerships at the different tiers within organizations and in particular the distinct challenges in organizations of varying size and financial stability. Inefficiency may be present when an organization is too narrowly focused or insufficiently focused. Inefficiency can result from running too many activities in a single program, or, as when there are unexploited economies of scope, too few (or in some manner leaving them inefficiently integrated) (*42*). Not surprisingly, the smaller organizations appeared to encounter fewer difficulties with intra-organizational communication, which was also enhanced by having personnel who worked across both the development and delivery arenas. Co-delivery of an inter-sectoral program also entails meeting the assorted expectations of managers, and challenges in finding common concepts and workplace language. Achieving this requires negotiation and time but is important for partnership cohesion and moving the partnership forward.

### Limitations

The practice of integrating health interventions and microfinance has progressed and many organizations have implemented such interventions. This review has evaluated only published literature and therefore has two limitations – publication bias and missing experiences of health-microfinance interventions that have not been published or exist as grey literature. We also had narrow inclusion criteria and therefore may have missed some studies. However, we intended to review only rigorously evaluated interventions to identify evidence-based strategies and therefore accepted these biases. As the focus of published studies was on the outcome and not organizational arrangements, many details are lacking in the structure, conditions, and governance that specifies how the organizational arrangements were developed and operationalized. Some authors do not provide sufficient evidence to distinguish between different types of organizational arrangements, so it was sometimes difficult to separate studies into our structured categories especially as boundaries were not clear on the partnership continuum.

## CONCLUSIONS AND POLICY IMPLICATIONS

This review identified the importance of multi-sectoral partnerships as an organizational strategy to build effective linkages of MFIs with the health sector. Coordination and collaboration partnerships are central to the delivery of services and have also been frequently cited as critical strategies for enhancing the effectiveness of health and human service systems (*43*). Public and nonprofit organizations as well as for-profit financial institutions, come together, often working across sectors, to address issues, solve problems, and provide services that are too complex, costly, and/or seemingly intractable for any one organization to handle on its own (*44*).

Given the increasing demands of multi-organizational partnerships to address complex problems, resources must be allocated to develop shared partnership processes and nurture partnership relations (*45*). A critical starting point is the growth of a successful microfinance operation that provides sustainable financial services to the poor (*46*) and sustainability must be able to induce efficiency improvement and better management practices (*47*). Past research has shown that enduring and high performing partnerships arise when both partners benefit equally from the relationship (*48*, *49*). Those stakeholders with the most immediate access to power and urgency are often partner organizations who control important resources or may provide access to important opportunities. Besides being central to partnership effectiveness, the maintenance of organization identity is necessary to partner commitment (*50*) and sustainability (*51*).

Despite calls for more robust evaluation frameworks with methodological innovations (*42*) to appraise partnership progress, there remain many challenges in doing so. In particular, there are well-described difficulties in attributing successful outcomes to partnership arrangements or determining whether observed benefits outweigh the costs of partnership (*45*). Most studies of collaboration are limited to the process of collaboration, its stages, or its success components (*52*). A study on the relationship between implementation structures and outcomes would be informative (*9*). On the other hand, public health studies have focused on health outcomes with minimal attention to organizational properties of MFIs and their partnership arrangements. Therefore, in approaching multidimensional, cross-sectoral partnership solutions, cross-disciplinary research holds a promise for advancing knowledge on how MFI and health partnerships can be strengthened and effectively scaled up. We need to explore and evaluate partnership models, functional linkages between the MFI and health sectors, and policy dialogue to include MFI-health interventions as a way of multiprogramming for addressing the economic and social determinants of health. Worldwide, health systems and micro-finance as separate programs are proving to be inadequate in meeting population needs. The global community could broaden its contribution to achieving health and social goals through multi-sectoral partnerships that utilize a microfinance platform to reach poor and underserved populations.
